# *Lactiplantibacillus plantarum* Induces Apoptosis in Melanoma and Breast Cancer Cells

**DOI:** 10.3390/microorganisms12010182

**Published:** 2024-01-17

**Authors:** Oana Budu, Alexandra Mioc, Codruta Soica, Florina Caruntu, Andreea Milan, Camelia Oprean, Daniel Lighezan, Slavita Rotunjanu, Viviana Ivan, Christian Banciu

**Affiliations:** 1 Faculty of Medicine, “Victor Babes” University of Medicine and Pharmacy, 2 Eftimie Murgu, 300041 Timisoara, Romania; oanadaniela.budu@yahoo.ro (O.B.); florina_caruntu@yahoo.com (F.C.); dlighezan@umft.ro (D.L.); ivan.viviana@umft.ro (V.I.); banciu.christian@umft.ro (C.B.); 2 Faculty of Pharmacy, “Victor Babes” University of Medicine and Pharmacy, 2 Eftimie Murgu, 300041 Timisoara, Romania; codrutasoica@umft.ro (C.S.); andreea.milan@umft.ro (A.M.); camelia.oprean@umft.ro (C.O.); slavita.rotunjanu@yahoo.com (S.R.); 3 OncoGen Centre, County Hospital ‘Pius Branzeu’, Blvd. Liviu Rebreanu 156, 300736 Timisoara, Romania

**Keywords:** *Lactiplantibacillus plantarum*, apoptosis, breast cancer, melanoma, cytotoxicity

## Abstract

Despite the notable advancements witnessed in the past decade in medical and health research domain, cancer remains a prominent global cause of mortality. Moreover, the conventional treatments employed to combat this disease have been found to considerably compromise the quality of life experienced by patients due to its severe side effects. Recent in vitro studies revealed encouraging findings on the potential beneficial effects of probiotics as adjuvants of anticancer therapy, and even as possible agents for the prevention and treatment of various types of malignancies. From this standpoint, the primary objective of this work was to investigate the anticancer properties of *Lactiplantibacillus plantarum* (LP) and elucidate its underlying mechanism of action. In order to investigate this matter, several doses of LP (ranging from 10^5^ to 10^10^ CFU/mL) were examined in relation to melanoma cancer cell lines (A375) and breast cancer cell line (MCF-7). The cell viability findings, which were substantiated by morphological investigations and annexin V/PI assay, indicated that LP exerted inhibitory effects on cellular activity and triggered apoptosis. Additionally, upon further investigation into its mechanism, it was observed through the apoptosis assay and Western blot analysis that the administration of LP resulted in an elevation of pro-apoptotic BAX protein levels and an upregulation of cleaved poly-ADP-ribose polymerase (PARP) protein expression. Conversely, the levels of anti-apoptotic Bcl-2 protein were found to decrease in the A375 and MCF-7 cell lines. These findings provide insight into the pro-apoptotic mechanism of action of LP in these specific cell lines.

## 1. Introduction

Probiotics have been identified as agents of medical significance due to their beneficial effects in preventing and supporting the treatment of severe chronic conditions such as cancer without inducing significant side effects [1]. The direct relationship between a Lactobacillus-enriched diet and a reduced incidence of colon cancer was reported in the 1980s, with several mechanisms being hypothesized such as the maintenance of physicochemical parameters of colon environment, and therefore homeostasis, as well as interspecies competition with putrefactive bacteria known to generate carcinogenic compounds [2]. The discovery of effective probiotic strains in clinical settings would have the potential to provide adjuvant treatment to anticancer drugs that have major side effects responsible for the decline in life quality and that generate drug resistance.

Probiotics are defined as live microorganisms lacking pathogenic potential able to provide health benefits for the host when administered in adequate quantities [3]; they are usually classified into bacterial strains and yeasts. Lactic acid-generating bacteria, such as *Lactobacillus* spp., have long been used in food industry, particularly fermented dairy products, where they improve final quality and preservation while also being internationally recognized for safe use [4]; their health benefits are due to either direct probiotic effects as a result of the interaction with the host or indirect biogenic effects through fermentation metabolites. *Lactobacillus* spp. are part of the human gut microbiota with large implication in metabolic and enzymatic processes, vital not only for the normal digestion, but also for the proper functioning of the immune system [5]. The most evident beneficial effects were at gastrointestinal level where lactobacilli significantly inhibit both precancerous [6] and cancerous lesions [7], and exert protective effects against inflammatory pathologies such as ulcerative colitis [8]. These findings were accompanied by the highlighting of immunomodulatory effects induced through the activation of several signaling pathways by probiotics’ metabolites such as short-chain fatty acids and extracellular proteins; as a result, an inhibition of genes associated with the tumor process occurs [9].

In breast cancer cells, probiotics may be used as prevention or as treatment due to their modulatory effect on gut bacteria as well as the immune system [10]; LP 5BL also acted directly against the proliferation of MCF-7 breast cancer cells while leaving healthy cells unharmed [10].

Heptelidic acid generating probiotics such as Aspergillus oryzae have the ability to inhibit the enzymatic activity of glyceraldehyde-3-phosphate dehydrogenase in B16F10 melanoma cells thus inhibiting their growth in a dose-dependent manner. Lipotechoic acid extracted from LP was able to inhibit melanogenesis in B16F10 cells by reducing the activity and expression of tyrosinase enzymes in a dose-dependent manner [11]. The tryptophan catabolite I3A synthesized by *L. reuteri* which is able to translocate and persist within melanoma induces the proliferation of CD8 cells and subsequently their production of interferon-γ; as a result, the catabolite promoted antitumor immunity and prolonged survival in melanoma patients [12]. Moreover, *L. reuteri* FLRE5K1 was able to stimulate the immune system in a murine model into producing anti-cancer cytokines and inhibiting the migration of melanoma B16F10 cells in order to prevent the onset of melanoma and prolong the overall mice survival [13].

However, since probiotic supplements were associated with diminished diversity of gut microbiome, which in turn has been linked to a poorer effect of the checkpoint inhibitor immunotherapy [14], there were unsurprisingly some controversial reports, while the bacteria-enriched gut microbiome in melanoma patients triggered a stronger response to anti-PD-1 treatment [15]. Another study revealed that probiotics impaired the antitumor effect of anti-PD-L1 treatment in melanoma-bearing mice that developed significant larger tumors compared to control [16]. The same study conducted an evaluation to determine if the utilization of probiotics exhibited any variations in outcomes among individuals undergoing immune checkpoint blockade treatment and revealed no statistically significant disparities in terms of progression-free survival between patients who reported probiotic intake and those who did not [16]. Therefore, the need to study the beneficial effects of probiotics still exists in order to further expand our knowledge on potential future anticancer therapies free from side effects.

The current study aimed to evaluate the antitumor effects of LP against melanoma cell lines (A375) and breast cancer cell line (MCF-7) in order to further reveal its underlying mechanisms; the study included in vitro studies such as the MTT assay, immunofluorescence staining, apoptosis, and Western blot assay.

## 2. Materials and Methods

### 2.1. Bacteria and Cell Lines

The bacterial strain LP was purchased from American Type Culture Collection (ATCC 8014, Łomianki, Poland), cultured in ATCC Medium 416: Lactobacilli MRS Agar/Broth and grown in aerobic conditions at 37 °C, in an atmosphere with 5% CO_2_. The bacterial strain suspension was centrifuged for 10 min at 3500 rpm and the optical density was adjusted at 600 nm (OD600) to obtain 2 × 10^10^ CFU/mL (colony-forming units per milliliter) (Microbiology Reader LogPhase 600, BioTek Instruments Inc., Winooski, VT, USA).

The cell lines used in this study were as follows: HaCaT immortalized human keratinocytes purchased from CLS Cell Lines ServiceGmbH (Eppelheim, Germany); A375 human malignant melanoma (ATCC^®^ CRL-1619TM) and MCF-7 human breast adenocarcinoma (ATCC^®^ HTB-22™) cell lines were purchased from ATTC American Type Culture Collection (Łomianki, Poland). HaCaT and A375 cell lines were cultured in Dulbecco’s Modified Eagle Medium (DMEM), while MCF-7 cell lines were cultured in Eagle’s Minimum Essential Medium (EMEM); all mediums were supplemented with 10% FBS (Thermo Fisher Scientific, Boston, MA, USA) and 1% penicillin/streptomycin mixture (10,000 IU/mL) (Thermo Fisher Scientific). The cells were grown under standard conditions in a humidified incubator with a 5% CO_2_ atmosphere at 37 °C and used for the experiments after reaching 80–85% confluency.

### 2.2. Cellular Viability Assessment

Cell viability was evaluated by means of MTT (3-(4,5-Dimethylthiazol-2-yl)-2,5-Diphenyltetrazolium Bromide) assay (Roche, Mannheim, Germany). The cells were cultured in 96-well plates at a density of 1 × 10^4^ cells/well. After reaching appropriate confluency, the cell culture media was discarded and changed with a mixture of antibiotic-free cell media and MRS broth to establish an environment supportive of both cellular and bacterial proliferation. The cells were then treated with six different concentrations of LP (10^5^–10^10^ CFU/mL). As a control measure, we examined the effect of LP media alone on cells, whereas 5-FU (10 μΜ) served as a positive control. The treated cells were maintained in aerobic conditions in a 5% CO_2_ atmosphere at 37 °C to mimic the physiological environment and support robust cell growth. Following the treatment period (48 h), the plates were incubated with 10 μL/well of MTT reagent for 3 h at 37 °C. This step was followed by the addition of 100 μL/well of MTT buffer solution and incubation for 30 min at room temperature and in the dark. Absorbance was measured at 570 nm using an xMark™ Microplate Spectrophotometer (Bio-Rad Laboratories, Inc., Hercules, CA, USA).

### 2.3. Chromatin Condensation

Evaluation of cellular morphology following treatment with various test compounds represent a reliable method to identify the type of induced cytotoxicity (i.e., cell death). The immunofluorescence assay was performed in HaCaT, A375, and MCF-7 cells treated for 48 h with the highest concentration of LP (10^10^ CFU/mL); the selection was based on cell viability results. The cells were labeled by Hoechst 33342 nuclear staining assay (Thermo Fisher Scientific) and Beta-Actin Mouse Monoclonal Antibody (Product MA5-15739, Thermo Fisher Scientific) [17]. The protocol applied for the immunofluorescence staining was established according to the manufacturer’s recommendation and adapted to our laboratory conditions, as follows: (i) treatment of cultured cells with 10^10^ CFU/mL LP for 48 h; (ii) cell fixation with 4% paraformaldehyde for 15 min; (iii) cell permeabilization with 0.25% Triton™ X-100 for 10 min, and blocking with 5% BSA for 1 h at room temperature; (iv) incubation with 2 µg/mL Beta-Actin Antibody in 0.1% BSA for 3 h at room temperature; (v) primary antibody labeling with 1:2000 Alexa Fluor 488 Goat Anti-Mouse Secondary Antibody (Product A28175, Thermo Fisher Scientific) for 45 min at room temperature; (vi) addition of Hoechst staining solution (5 μg/mL), and 10 min incubation at room temperature and in the dark; (vii) 3x washing steps with PBS. The images were acquired using the CellSens Entry Software version 3.0 (Olympus, Tokyo, Japan), DP28-CU camera (Olympus), and the inverted fluorescent microscope Optika IM-3FL4 (Optika, Ponteranica, Italy).

### 2.4. Annexin-V-FITC/Propidium Iodide (PI) Staining

The evaluation of cell death induced by LP (10^10^ CFU/mL) in A375 and MCF-7 cells was conducted through the annexin V/PI method [18]. A375 and MCF-7 cells were cultured in 6-well plates at a density of 1 × 10^6^ cells/well and allowed to attach for 24 h. Subsequently, the culture medium was replaced with a fresh medium containing the sample at final concentrations (10^10^ CFU/mL). Untreated cells served as control. After 48 h of incubation, cells were trypsinized, and Annexin V-FITC combined with PI from the Annexin V-FITC kit (Invitrogen, ThermoFisher, Vienna, Austria) was used for flow cytometric analysis of cell death (apoptosis and necrosis). Following detachment, cells were washed twice with the buffer solution 1 × Annexin V Binding buffer, resuspended in 195 μL Binding buffer and incubated with Annexin V-FITC in the dark for 15 min. After incubation, cells were washed again, resuspended in 190 μL Binding buffer, and PI solution was added 10 min before flow cytometric analysis. Data were acquired using a FACSCalibur flow cytometer (Becton Dickinson, Franklin Lakes, NJ, USA).

### 2.5. BAX and Bcl-2 Detection

The activities of pro-apoptotic BAX and anti-apoptotic Bcl-2 proteins were measured by colorimetric assay kits (ab199080 and ab119506; Abcam plc, Cambridge, UK). HaCaT, A375, and MCF-7 cells were cultured in a 6-well plate and treated for 48 h with 10^10^ CFU/mL LP. The level of BAX and Bcl-2 was determined following the assay procedure described in the manufacturer’s instructions [19]. The total protein concentration was determined using the Pierce™ Rapid Gold BCA Protein Assay Kit (Thermo Fisher Scientific). The absorbance was read at 450 nm using an xMark™ Microplate Spectrophotometer (Bio-Rad, Hercules, CA, USA).

### 2.6. Western Blot

HaCaT, A375, and MCF-7 cell lines were cultured in a 6-well plate at a density of 1 × 10^6^ cells/well. After 24 h, the cells were treated with LP 10^10^ CFU/mL and incubated for another 48 h at 37 °C. The Western blot workflow was performed following the recommendations of Pillai-Kastoori et al. [20]. After treatment, cells were removed and lyzed in RIPA buffer (Thermo Fisher Scientific). Protein concentration was measured according to the manufacturer’s protocol using the Pierce™ Rapid Gold BCA Protein Assay Kit (Thermo Fisher Scientific) [21]. The Western blot analysis was performed by loading equal amounts of proteins (20 μg) in each lane of a Novex^®^ NuPAGE^®^ 4–12% Bis-Tris gel (NP0321BOX, Thermo Fisher Scientific). Following separation (Mini Gel Tank-A25977, Thermo Fisher Scientific), the proteins were transferred on a nitro-cellulose membrane by iBlot^®^ 2 Dry Blotting System (IB23001, Thermo Fisher Scientific) and the membranes obtained were blocked with 5% skimmed milk. The blots were probed with 1:1000 Anti-PARP1 (cleaved Asp214) monoclonal antibody (14-6668-82, Thermo Fisher Scientific) in blocking buffer at 4 °C, overnight. Goat anti-Mouse IgG (H+L) Superclonal™ Recombinant Secondary Antibody, HRP (1:2000 dilution, Thermo Fisher Scientific) was used as a secondary antibody, while beta-tubulin Mouse Monoclonal Antibody 1:1000 dilution (Product # 32-2600, Thermo Fisher Scientific) was used as a loading control. The chemiluminescent detection was performed using Pierce™ ECL Western blotting substrate (Thermo Fisher Scientific) in a ChemiDoc MP Imaging System (170-8280). Image acquisition and analysis were achieved using the Image Lab software version 6.1 (BioRad).

### 2.7. Statistical Analysis

The statistical analysis was performed using one-way ANOVA followed by Bonferroni’s multiple comparisons post-test (GraphPad Prism version 6.0.0, GraphPad Software, San Diego, CA, USA). The differences between groups were considered statistically significant if *p* < 0.05, as follows: * *p* < 0.05, ** *p* < 0.01, and *** *p* < 0.001.

## 3. Results

### 3.1. Assessment of LP Cytotoxic Activity

**Figure 1:**
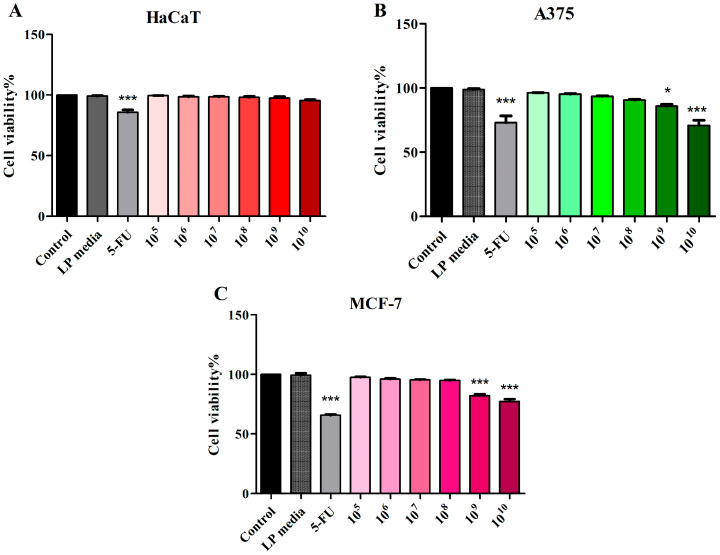
Cell viability of HaCaT (**A**), A375 (**B**), and MCF-7 (**C**) cells following 48 h treatment with LP 10^5^–10^10^ CFU/mL and 5-FU (10 μΜ). The results are expressed as viability percentage in comparison with the control group, considered 100% (* *p* < 0.05 and *** *p* < 0.001). The data represent the mean values ± SD of three independent experiments performed in triplicate.

The LP (10^5^–10^10^ CFU/mL) cytotoxic effect on nonmalignant HaCaT, malignant melanoma A375, and breast adenocarcinoma MCF-7 cell lines was evaluated using the MTT assay. The LP media did not influence cell viability of any cell line used in this study (Figure 1). No significant changes in cellular viability were detected in normal HaCaT cell lines treated with 10^5^–10^10^ CFU/mL LP vs. control (Figure 1A). A375 melanoma cells treated with 10^10^ and 10^9^ CFU/mL LP exhibited a significantly decreased cellular viability (70.86 ± 6.91 and 85.68 ± 2.60) vs. control (100%). The positive control, 5-FU (10 μM) decreased cell viability to 72.13 ± 7.07 vs. control (Figure 1B). LP treatment on MCF-7 breast adenocarcinoma was able to decrease cell viability only when tested at 10^10^ and 10^9^ CFU/mL (77.34 ± 3.44 and 82.11 ± 2.31) vs. control (100%) and vs. 5-FU (65.83 ± 1.35) (Figure 1C).

In nonmalignant HaCaT cell line, no significant morphological changes were observed between the control group and the LP 10^8^–10^10^ CFU/mL-treated cells, in terms of confluence; few detached cells were observed in the LP 10^10^ and 10^9^ CFU/mL-treated cells, but they did not influence the cellular viability in a significant manner. 5-FU was used as a positive control. Compared to 5-FU and control, LP treatment does not significantly influence the cellular morphology of normal HaCaT cells (Figure S1). Upon evaluation of A375 cellular morphology, LP treatment decreased the number of adherent cells, producing cell detachment and an effect directly proportional with the concentration used (Figure S2).

LP treatment with 10^9^ and 10^10^ CFU/mL on MCF-7 decreased the number of cells and altered the cellular morphology, rendering them detached. The morphology and shape of LP 10^8^ CFU/mL-treated cancer cells were similar to that of control cells: adherent and confluent cells (Figure S3).

### 3.2. Evaluation of Apoptotic Features by Immunofluorescent Staining

**Figure 2:**
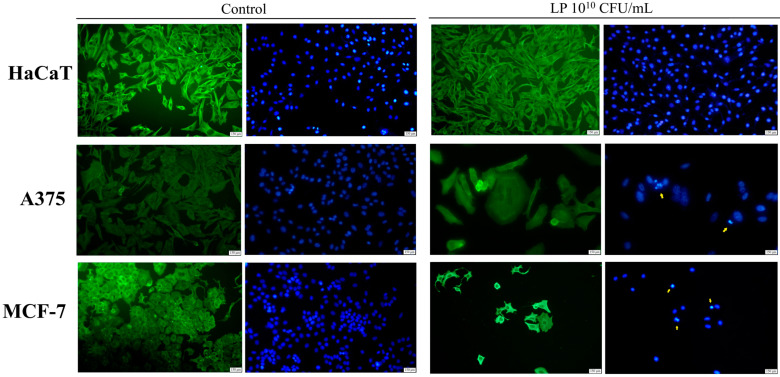
The impact of 48 h treatment with 10^10^ CFU/mL LP on HaCaT, A375, and MCF-7 cell lines; beta-actin—green staining and nuclei—blue staining. The yellow arrows indicate signs of apoptosis.

The effect of LP (10^10^ CFU/mL) on the morphology of HaCaT, A375, and MCF-7 cells was also investigated using the Hoechst and beta-actin/Alexa Fluor 488 immunofluorescent staining (Figure 2). LP treatment in the highest concentration on HaCaT cells did not induce any significant morphological alterations (Figure 2). However, in A375 cell lines, LP treatment at the same concentration was able to disrupt cell cytoskeleton and to induce nuclear fragmentation and condensation, features that are consistent with apoptotic cell death (Figure 2). MCF-7 cells treated with 10^10^ CFU/mL LP exhibited similar changes in cellular morphology consistent with cell death by apoptosis. Specifically, treated MCF-7 cells showed nuclear condensation, fragmentation, and membrane blebbing and cytoskeleton disorganization (Figure 2). When comparing the distribution of the actin filaments between control and the treated group of each cancer cell line, one can observe that β-actin is proportional and is concentrated in the whole cell body of the cell dispersion, whereas in the treated cancer cells, its distribution is concentrated mainly in the cortical ring found under the cell membrane (Figure 2).

### 3.3. Annexin V/PI Analysis

**Figure 3:**
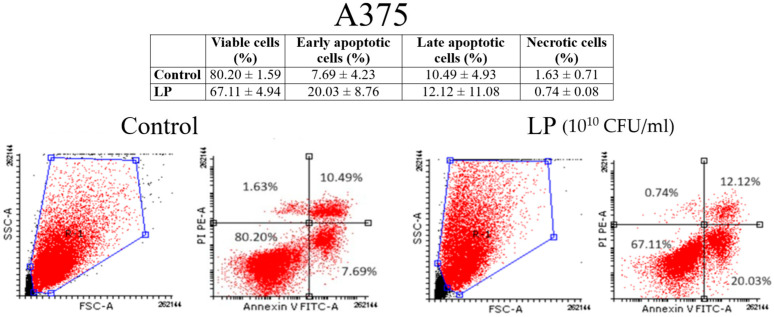
Identification of apoptosis in A375 cells after (LP 10^10^ CFU/mL) by the annexin V/PI double staining: viable cells (**left bottom**), early apoptotic cells (**right bottom**), late apoptotic cells (**right top**), and necrotic cells (**left top**).

**Figure 4:**
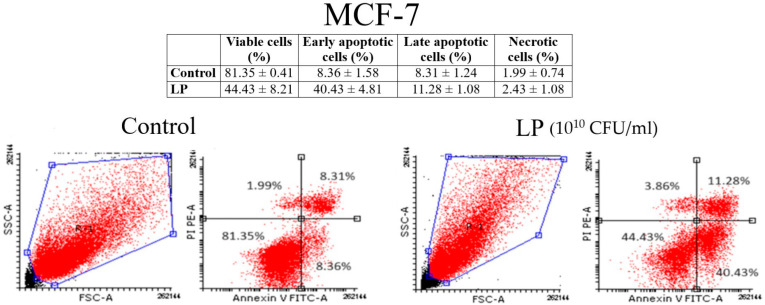
Identification of apoptosis in MCF-7 cells after (LP 10^10^ CFU/mL) by the annexin V/PI double staining: viable cells (**left bottom**), early apoptotic cells (**right bottom**), late apoptotic cells (**right top**), and necrotic cells (**left top**).

Figure 3 and Figure 4 show the results of annexin V/PI analysis on A375 and MCF-7 cells. One can notice that LP (10^10^ CFU/mL) induced the apoptosis both in A375 (Figure 3) and MCF-7 cells (Figure 4). In the case of A375 cells, the percentage of the early apoptotic cells of the untreated cells was 7.69 ± 4.23%, while the percentage of the early apoptotic cells of the cells treated for 48 h with the LP was 20.03 ± 8.76%. As for the MCF-7 cells, the percentage of early apoptotic cells grew from 8.36 ± 1.58% (untreated cells) to 40.43 ± 4.81% (cells treated with LP).

### 3.4. Apoptosis Assay—LP Effect on BAX and Bcl-2 Protein Levels

**Figure 5:**
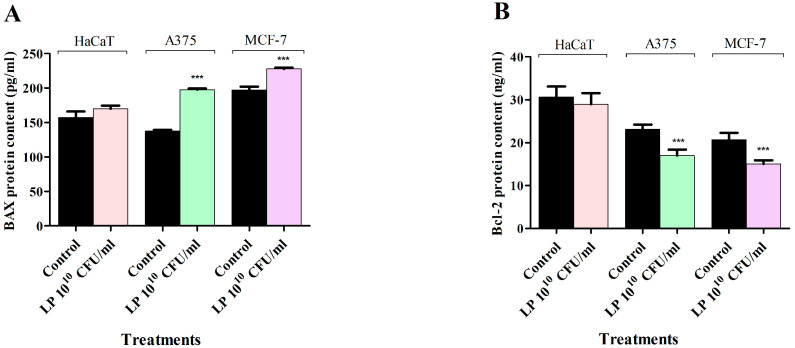
Effect of 10^10^ CFU/mL LP on BAX (**A**) and Bcl-2 (**B**) protein levels in HaCaT, A375, and MCF-7 cell lines after 48 h treatment. The results were reported as mean values ± SD with *p* < 0.001 (***) when compared to their respective control. All experiments were performed in triplicate.

In order to determine whether LP treatment induces apoptotic cell death in HaCaT, A375, and MCF-7 cancer cells, the pro-apoptotic BAX and anti-apoptotic Bcl-2 levels were quantitatively measured using an in vitro enzyme-linked immunosorbent assay (ELISA). The results indicate that LP treatment can increase the pro-apoptotic BAX protein level, while decreasing the anti-apoptotic Bcl-2 protein level in all cancer cell lines (Figure 5). In normal cells, LP treatment had a similar tendency in changing the BAX and Bcl-2 protein levels; however, these effects did not reach statistical significance (Figure 5).

### 3.5. Western Blot Analysis

**Figure 6:**
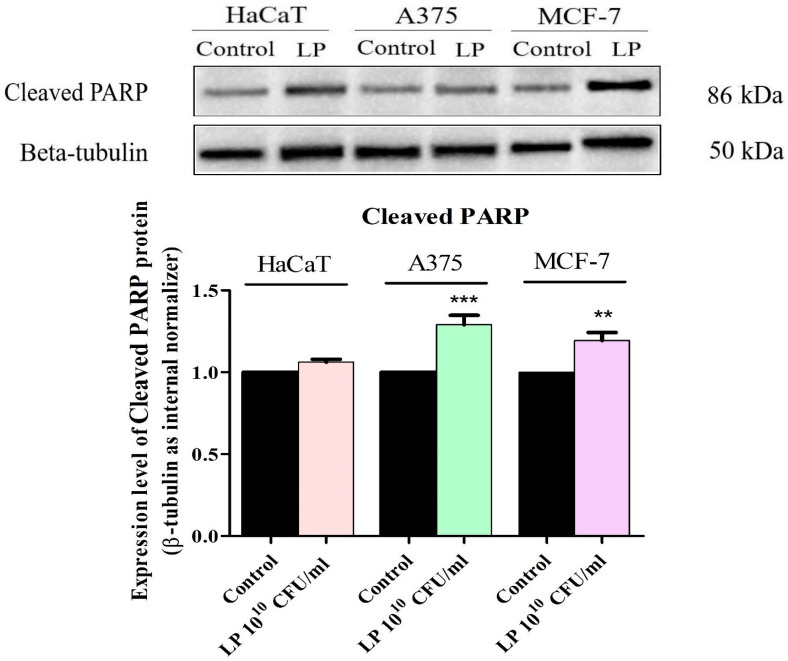
Determination of cleaved PARP protein expression in HaCaT, A375, and MCF-7 cells. The results were normalized against the beta-tubulin loading control and the control group. The statistical differences vs. control were determined using one-way ANOVA analysis followed by Tukey’s multiple comparisons post-test ( ** *p* < 0.01, and *** *p* < 0.001).

In order to clearly establish the cytotoxic mechanism of LP, the protein expression levels of cleaved PARP, a marker of apoptotic cell death, were determined by Western blot (Figure 6). Upon treatment with LP 10^10^ CFU/mL, expression of cleaved PARP was upregulated in all cancer cell lines (Figure 6). No statistically significant increase in cleaved PARP level was detected in normal HaCaT cells after LP treatment (Figure 6).

## 4. Discussion

Cancer is a leading cause of death worldwide and may affect any organ in the body; according to WHO, breast cancer was the most common type of cancer with 2.26 million cases in 2020 [22]. Also, skin melanoma represents 1.7% of global cancer cases with approximately 325,000 new cases in 2020 [23]. Conventional treatments come with severe side effects that diminish the quality of life or cause drug resistance thus impairing patient’s survival. Therefore, any new adjuvant or alternative aiding or providing a selective and effective anticancer treatment would be appealing to both cancer patients and health professionals.

Probiotics have long been associated with health benefits and were included in the last decade as adjuvants against neoplastic pathologies; LP belongs to the widely used lactic acid bacteria able to generate various metabolites with apoptotic, antimutagenic, and antimetastatic potential [24].

LP was tested against breast cancer and melanoma cells in order to identify its potential anticancer mechanisms which would enable future treatment options with weaker or no side effects; the range of LP tested concentrations was selected according to previously published studies. As an example, in vitro and in vivo studies of *L. reuterii* against B16F10 melanoma cells were conducted using 10^7^ CFU bacterial suspension; the authors indicated that a minimal concentration of 10^6^ CFU/mL viable bacteria is necessary for probiotic activity [13]. In addition, concentrations of 10^8^–10^9^ CFU/dose or higher are found in commercial products advertised with probiotic effects [25]. Molska and Regula reported that, although the optimal concentration of probiotics that should be recommended for cancer treatment or prevention is not yet clearly established, it cannot be low if a successful impact on the host organism is to be expected; the authors mentioned that 10^10^–10^11^ CFU/day of *Lactobacillus* spp. are needed for colon cancer prevention [26].

The cytotoxic activity in melanoma cells was comparable to the one recorded for the anticancer conventional drug 5-FU when LP was used in concentrations of 10^10^ and 10^9^ CFU/mL; similar concentrations of *L. rhamnosus* selectively reduced viability in A375 melanoma cells in a concentration-dependent manner [27]. 5-FU is usually associated with mucositis, diarrhea, myelosuppression, cardio- and neurotoxicity that make systemic treatment difficult for cancer patients; the use of LP may therefore replace a highly toxic anticancer therapy with a more acceptable one in terms of quality of life for the treated patients. Our results are in line with the results reported in 2020 by Park et al. in A375 melanoma cells where LP extract exerted a strong antiproliferative activity, in particular in high metastatic A375 cells accompanied by an anti-migratory effect [28]. LP anti-melanoma potential effect was confirmed in vivo where a significant decrease in the serum levels of angiogenic VEGF and a significant increase in the serum levels of immunostimulatory IL-12 were recorded [29]. In breast cancer cells, LP extract exerted a similar antiproliferative activity to the one recorded against melanoma cells and comparable to the conventional 5-FU; this behavior was to be expected since LP revealed improved clinical and laboratory profiles, in particular WBC level, in breast cancer patients who received LP as adjuvant therapy in combination with conventional drugs [30]. Indeed, various LP strains induced cytotoxic effects against different cancer cell lines, including MCF-7, where the UL4 strain revealed the lowest IC_50_ value combined with high selectivity against cancer cells; the authors concluded that LP extracts exert their selective antiproliferative effects in a strain-specific and cancer cell type-specific manner [31]. Although in vivo probiotic bacteria do not come in direct contact with breast cancer cells, their beneficial effect in reducing tumor frequency was undisputably revealed and attributed to systemic immunomodulatory effects induced by LP in tumor tissue such as increased levels of CD4+T- and CD8+T-cells as well as reduced serum TNF concentrations [32]. In vitro studies emphasized that the cytotoxic activity of LP against MCF-7 breast cancer cells may be attributed to proteinaceous postbiotic metabolites that act in a time- and dose-dependent manner [33]; corroborating in vitro and in vivo data, one can only assume that such metabolites as well as others can be absorbed after probiotics’ oral administration and reach tumor cells in the vivo mechanism that combines with immunomodulatory systemic activity in producing an overall anticancer effect. Another possibility was very recently introduced by Bender et al. who showed that viable *L. reuteri* is able to translocate from the gut to melanoma tumors where it triggers antitumor Tc1 immunity through its production of tryptophan catabolite indole-3-aldehyde. The antitumor effect was also reported after injecting viable bacteria into the melanoma tumor; the authors concluded that the intratumoral presence of viable *L. reuteri* is required to promote immunomodulatory effects as well as suppress tumor growth, such effects strongly depend on its metabolic activity [12]. In light of these findings, it is worth mentioning that despite older beliefs, the breast tissue, both normal and malignant, also possesses a specific microbiota; normal microbiome is not only involved in maintaining the health of breast tissue through the stimulation of resident immune cells, but also through its ability to degrade potential carcinogens [34]. Distinct microbial patterns characterize malignant breast tissue with different bacterial loads and diversity for each type of breast cancer; moreover, in breast cancer, gut microbiota displays significant changes in terms of compositional bacterial abundance as well as microbial metabolites [35].

An important aspect of LP’s cytotoxic activity is its selectivity as revealed by the lack of cell death regardless of the applied concentration when the MTT assay was conducted in HaCaT cells (immortalized nonmalignant keratinocytes), a line widely used to study the multistage development of cancer in human cells as well as epidermal pathophysiology and homeostasis. The cell viability results were confirmed by morphological studies that indicated a decreased number of adherent cells in both cancer lines in a dose-dependent manner simultaneously with no significant morphological changes in HaCaT keratinocytes.

The underlying molecular mechanism of LP cytotoxic effect was studied by means of immunofluorescence staining that revealed the disruption of the cytoskeleton resulting in the alteration of the cell’s normal architecture as well as nuclear fragmentation and condensation. Beta-actin is a crucial component of cell cytoskeleton that has the ability to quickly form or dismantle filaments according to the cellular demands. Moreover, studies revealed that beta-actin is not only a scaffold for cell cytoskeleton, but also an apoptosis modulator; in vascular smooth muscle cells, the disruption of beta-actin cytoskeleton led to the release of Bmf pro-apoptotic marker, and consequently to apoptosis [36]. The correct position within the cell of essential cell components depends on the direction and integrity of cytoskeleton filaments [37]. Therefore, cytoskeleton disorganization together with nuclear shrinkage is able to induce cell death, which is being regarded as a sign of apoptosis. Indeed, linoleic acid produced through metabolism by LP was shown to induce apoptosis in breast cancer cells [24], while LP extract induced intrinsic apoptosis in A375 melanoma cells simultaneously inhibiting their migration ability [28]. Studies have shown that tumor microbiota plays a significant role in the survival of metastatic tumor cells of various types through reorganizing actin cytoskeleton, and thus enhancing their resistance to fluid shear stress [38]; administration of probiotics that have the ability to cause cytoskeleton disruption might therefore not only alter the tumor microbiome, but also reduce the survival of circulating tumor cells responsible for distant metastasis. As an example, probiotics contained in kefir exhibited antimetastatic as well as antiangiogenic effects in mouse breast cancer cells [39]. The immunofluorescence staining results were confirmed by the dual annexin V/PI staining flow cytometry, used to assess the apoptosis status of melanoma A375 and breast cancer MCF-7 cells; the results showed that LP treatment was able to increase the percentage of early apoptotic cells compared to control. All apoptotic tests were conducted by using the highest effective concentration, 10^10^ CFU/mL, based on the dose-dependent antiproliferative effect and in order to distinctly identify the apoptotic effect versus a potential necrotic effect that might have been induced physically upon cancer cells by the high-density bacterial population present at such concentration. The results can be extrapolated to smaller concentrations where the bacterial density diminishes and the apoptotic effects clearly prevail.

The induction of apoptosis in cancer cells represents the most efficient way to treat cancer. Our study continued with a deeper investigation of the pro- (BAX) and anti-apoptotic (Bcl-2) protein levels; the Bcl-2 protein family comprises members with either pro- or anti-apoptotic properties that have been extensively researched for their role in regulating apoptosis, carcinogenesis, and cell response to anticancer treatment. The mitochondria-mediated intrinsic apoptosis regulated by the Bcl-2 protein family and their effector caspases induce the degradation phase of apoptosis described above, that includes disruption of the cell architecture, nuclear condensation, DNA fragmentation, and membrane blebbing [40]. In our study, upon treatment with LP 10^10^ CFU/mL the protein level of Bcl-2 was decreased, whereas BAX levels were increased in all three cancer cell lines, thus revealing one molecular mechanism for the apoptosis of the tested cancer cells. These experimental results are confirmed by previous literature reports; Ardestani et al. revealed that *Lactobacillus brevis* inhibited the growth of HT-29 colon cancer cells and induced apoptosis by increasing the expression of BAX, caspase-3, and caspase-9 mRNA levels, while reducing the expression of Bcl-2 protein levels [41]. Indeed, LP extract was previously reported to decrease the level of the anti-apoptotic Bcl-2 protein and increase the apoptotic BAX protein, simultaneously activating caspase-3 and -9 [28], thus qualifying as an intrinsic apoptosis inducer; similarly, Senturk et al. revealed the downregulation of Bcl-2 in MCF-7 breast cancer cells by LP secondary metabolites [42]. Controversially, an opposite effect was reported in 2015 by Kim et al. who identified an inhibitory effect of LP extracts on the apoptosis of HT-29 colon cancer cells by downregulating the generation of caspase-9 and -3 and activating the expression of Bcl-2 [43]. However, their experiment involved the administration of LP extracts in colon cancer cells previously infected with Staphylococcus aureus able to induce cell apoptosis in order to diffuse to other cells. Moreover, the study revealed that LP extract-treated cells had an increased Bcl-2 expression, compared with the S. aureus only cells, while the peptidoglycan and lipoteichoic acid of LP inhibited the apoptosis mediated by *S. aureus* [43]. In another study, rats were exposed to whole-body Gamma-ray radiation and were gavaged with 0.2 mL of 1 × 10^10^ *Lactobacillus* spp. for 4 weeks, and then their testicular tissue was analyzed using the quantitative real-time polymerase chain reaction (qRT-PCR). Similarly, this study revealed that Gamma-radiation inhibited Bcl-2 and activated BAX and caspase-3 genes in healthy animal tissues, and *Lactiplantibacillus* spp. treatment was able to reverse such effects and acted as natural radioprotectors [44]. On the other hand, LP 06CC2 extract significantly suppressed cell viability of Caco2 colorectal cancer cells by inducing apoptosis through the JNK/p38 MAPK signaling system [45]. In addition, Caco2 colorectal cancer cells were sensitive to the LP metabolites that were able produce a notable reduction in colon cancer cells viability following a relative short treatment duration (6–24 h), and even at 10% dilution. However, the authors reported that the cytotoxic mechanism of LP metabolites involves cell autophagy rather than apoptosis; the LP metabolites impeded the process of phagophore and autophagosome formation, thus leading to a reduction in the expression of autophagy proteins Atg9A, LC3 I/II, Atg5, Atg16L1, and Beclin-1 [46]. All data considered, one may conclude that in healthy cells as well as in cells exposed to previous aggression such as S. aureus infection, LP acts as an apoptosis inhibitor while in cancer cells the effect is reversed; these opposite effects take place based on the same mechanism, the inhibition or activation of apoptosis-related genes, which evolves in opposite directions depending on the cell type.

As described above, the Bcl-2 family protein and the caspase effectors, especially caspase-3, regulate the apoptotic cell death. One of the main targets of caspase-3, and implicitly, Bcl-2 family protein, is nuclear poly (ADP-ribose) polymerase (PARP), a protein involved in DNA repair [47,48]. Cleavage of PARP by caspase-3 occurs during the apoptotic program to prevent DNA repair, and thus cleaved PARP serves as a marker of cells undergoing apoptosis [47,48]. Considering the results obtained after the nuclear morphological assessment and the apoptosis assay results, a more in-depth analysis to confirm the apoptotic mechanism of action of LP was performed by evaluating the cleaved PARP protein expression by Western blot analysis. The results clearly show that LP treatment increases the expression level of cleaved PARP in all cancer cell lines. Similar results were described by Kim et al. who reported that a *Lactobacillus rhamnosus* derived protein (p8) increased cleaved PARP1 expression and led to apoptotic cells in a mouse xenograft model of colorectal cancer [49]. Moreover, the cytotoxic and pro-apoptotic effects of *Bacillus coagulans* on COLO205 colon adenocarcinoma cell line was also attributed to increased BAX/Bcl-2 ratio, increased caspase-3 levels and PARP cleavage [50]. In our study, LP did not significantly modify the BAX/Bcl-2 ratio, nor the cleaved PARP expression level in HaCaT cells, thus revealing a selective activity on cancer cells. Further studies will be needed in order to properly assess the therapeutic potential of LP extracts against cancer.

Future studies are needed in order to clarify in vivo aspects of the therapeutical benefits provided by the administration of probiotics in breast cancer and malignant melanoma; however, one can expect promising results since some previous papers reported some very effective in vivo results. As an example, a review published in 2020 by Eslami et al. stated that the microbiome represents a risk factor for breast cancer and is essential for its response to therapy [51]; the authors also reported various species of *Lactobacillus* able to inhibit early-stage breast carcinogenesis and to induce tumor reduction. Similarly, Thu et al. collected data on randomized clinical trials regarding the use of probiotics in breast cancer patients and survivors; their systematic review and meta-analysis revealed that probiotics reduced both cancer severity and symptoms leading to improved prognosis [52]. LP was administered to experimental melanoma-bearing mice where it acted as an anticancer agent through the stimulation of the immune system as well as angiogenesis suppression [29]. Another species, *L. reuteri*, inhibited in vivo the migration of B16F10 melanoma cells, thus preventing melanoma onset and prolonging mice survival [13]. In light of these findings, future in vivo studies will complete the overall image of the therapeutic effects of probiotics in general and LP, in particular against melanoma and breast cancer.

## 5. Conclusions

In the current study, LP was investigated in terms of its antiproliferative activity against two melanoma and one breast cancer cell lines revealing a significant cytotoxic activity, comparable to the conventional anticancer drug 5-FU. These findings prompted further investigation that showed LP ability to disrupt the cell cytoskeleton, alter nuclei morphology, and induce apoptosis. The Western blot analysis revealed upregulated levels of cleaved PARP which, associated with the increase in BAX and decrease in Bcl-2 protein levels, support the pro-apoptotic mechanism of action; in addition, LP exhibited minimal effects in normal HaCaT cells that might indicate a selective activity. Further studies are necessary in order to fully establish its adjuvant potential in anticancer treatments. Numerous studies have demonstrated the potential benefits of probiotic administration in preventing cancer and enhancing anti-cancer treatments. Furthermore, encouraging findings have emerged and suggested that probiotics have anticancer effects. The research findings reported, however, only support the usefulness of probiotics as a possible cancer preventive measure or as an adjuvant therapy in conjunction with traditional anticancer chemotherapy. As a future perspective with regard to probiotics and cancer treatment, in order to definitively confirm the probiotic’s potential in this area, more studies on the anti-cancer mechanisms of action of particular probiotic strains as well as clinical investigations are mandatory to obtain the medical community approval and to confirm the efficacy of probiotics as a possible option for cancer treatment.

## Data Availability

The original contributions presented in the study are included in the article/Supplementary Material, further inquiries can be directed to the corresponding author/s.

## Supplementary Materials

The following supporting information can be downloaded at https://www.mdpi.com/article/10.3390/microorganisms12010182/s1. Figure S1: The evaluation of morphological changes in HaCaT cells after 48 h treatment with 10^8^, 10^9^, 10^10^ CFU/mL LP and 10 μΜ 5-FU; the scale bar was 150 μm. Figure S2: The evaluation of morphological changes in A375 cells after 48 h treatment with 10^8^, 10^9^, 10^10^ CFU/mL LP and 10 μΜ 5-FU; the scale bar was 150 μm. Figure S3: The evaluation of morphological changes in MCF-7 cells after 48 h treatment with 10^8^, 10^9^, 10^10^ CFU/mL LP and 10 μΜ 5-FU; the scale bar was 150 μm.
