# Supplementary material for: Lactiplantibacillus plantarum Induces Apoptosis in Melanoma and Breast Cancer Cells
Source: Microorganisms. 2024 Jan 17;12(1):182. doi: 10.3390/microorganisms12010182 (PMC10819835; doi:10.3390/microorganisms12010182)
Supplement: Supplementary file 1 [file microorganisms-12-00182-s001.zip › microorganisms-2825041-Supplementary.pdf]

microorganisms

MDPI

Supplementary Material

Lactiplantibacillus plantarum induces apoptosis in melanoma and breast cancer cells

Oana Budu[1], Alexandra Mioc[2,*], Codruta Soica[2], Florina Caruntu[1], Andreea Milan[2], Camelia Oprean[2,3], Daniel Lighezan[1], Slavita Rotunjanu[2], Viviana Ivan[1], Christian Banciu[1]

*** Correspondence:** Corresponding Author: alexandra.mioc@umft.ro

1.1. Supplementary Figures

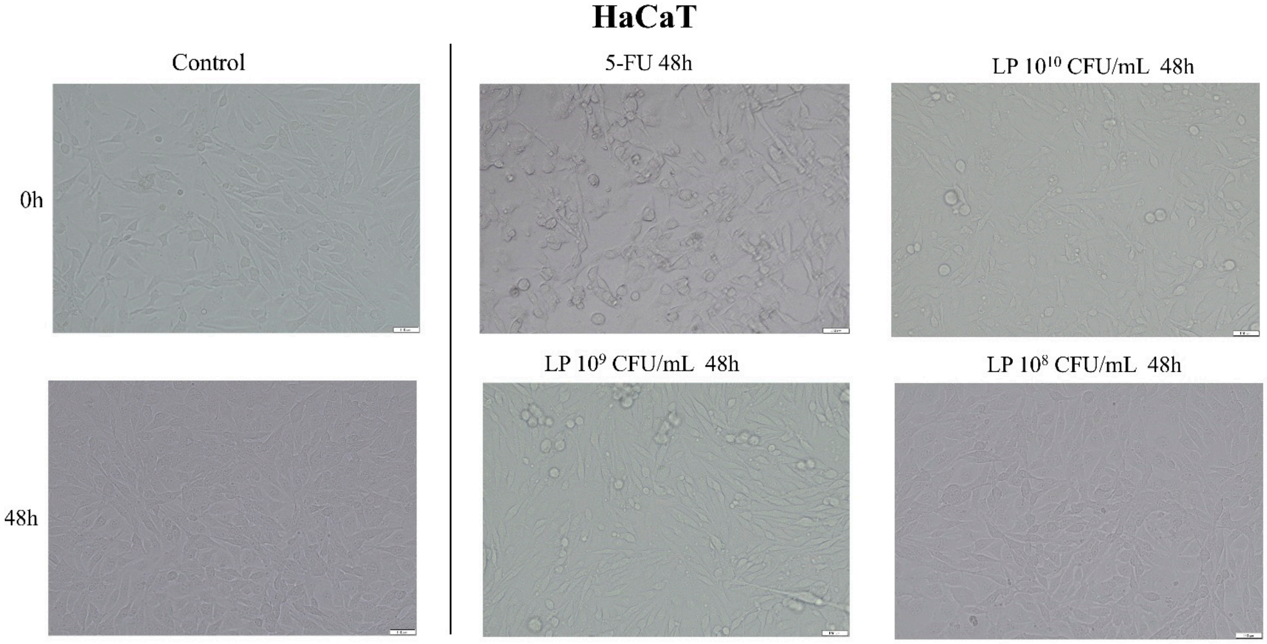

Supplementary Figure S1. The evaluation of morphological changes of HaCaT cells after 48 h treatment with 10^8, 10^9, 10^{10} CFU/ml LP and 10 μM 5-FU; the scale bar was 150 μm.

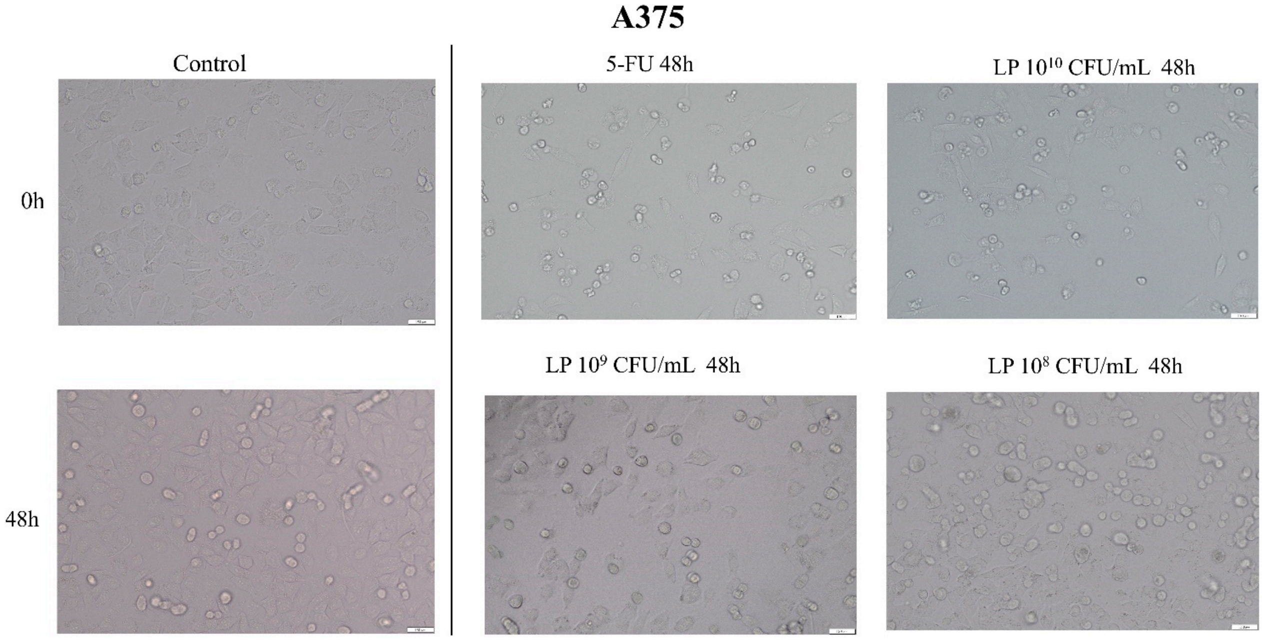

Supplementary Figure S2. The evaluation of morphological changes of A375 cells after 48 h treatment with 108, 109, 1010 CFU/ml LP and 10 μM 5-FU; the scale bar was 150 μm.

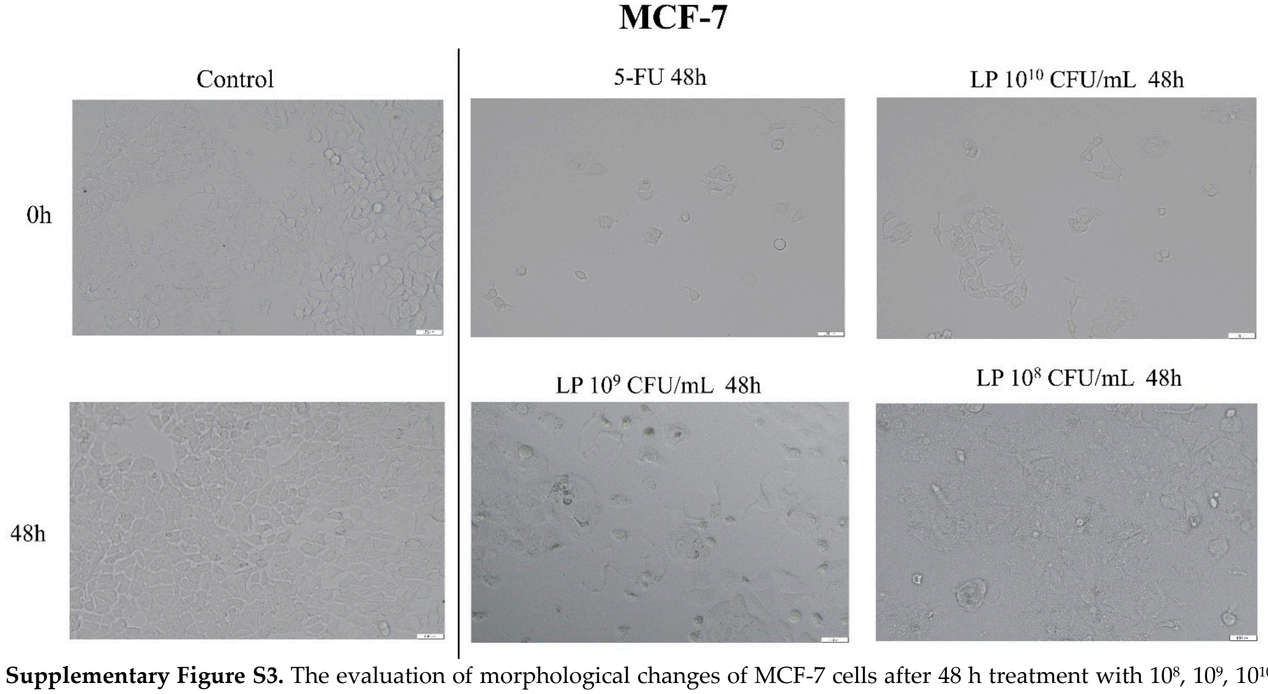

Supplementary Figure S3. The evaluation of morphological changes of MCF-7 cells after 48 h treatment with 10^8, 10^9, 10^{10} CFU/ml LP and 10 μM 5-FU; the scale bar was 150 μm.
